# Expression Patterns of Genes Involved in the Defense and Stress Response of *Spiroplasma citri* Infected Madagascar Periwinkle *Catharanthus roseus*

**DOI:** 10.3390/ijms13022301

**Published:** 2012-02-21

**Authors:** Naghmeh Nejat, Ganesan Vadamalai, Matthew Dickinson

**Affiliations:** 1 Institute of Tropical Agriculture, University Putra Malaysia, Serdang 43400, Malaysia; 2 Plant Protection Department, Faculty of Agriculture, University Putra Malaysia, Serdang 43400, Malaysia; E-Mail: ganesanv@putra.upm.edu.my; 3 School of Biosciences, University of Nottingham, Sutton Bonington Campus, Loughborough LE12 5RD, UK; E-Mail: matthew.dickinson@nottingham.ac.uk

**Keywords:** quantitative Real-time PCR (RT-qPCR), metallothionein, heat shock protein 90, extensin, strictosidine β-glucosidase

## Abstract

Madagascar periwinkle is an ornamental and a medicinal plant, and is also an indicator plant that is highly susceptible to phytoplasma and spiroplasma infections from different crops. Periwinkle lethal yellows, caused by *Spiroplasma citri*, is one of the most devastating diseases of periwinkle. The response of plants to *S. citri* infection is very little known at the transcriptome level. In this study, quantitative real-time PCR (RT-qPCR) was used to investigate the expression levels of four selected genes involved in defense and stress responses in naturally and experimentally *Spiroplasma citri* infected periwinkles. Strictosidine β-glucosidase involved in terpenoid indole alkaloids (TIAs) biosynthesis pathway showed significant upregulation in experimentally and naturally infected periwinkles. The transcript level of extensin increased in leaves of periwinkles experimentally infected by *S. citri* in comparison to healthy ones. A similar level of heat shock protein 90 and metallothionein expression was observed in healthy, naturally and experimentally spiroplasma-diseased periwinkles. Overexpression of Strictosidine β-glucosidase demonstrates the potential utility of this gene as a host biomarker to increase the fidelity of *S. citri* detection and can also be used in breeding programs to develop stable disease-resistance varieties.

## 1. Introduction

*Catharanthus roseus* (L.) G. Don, known as the Madagascar periwinkle which belongs to the Apocynaceae family is one of the few medicinal plants that was mentioned in the folk medicinal literature as early as 2 BC, and is known to produce a large number of pharmaceutically valuable dimeric terpenoid indole alkaloids which are used in the treatment of hypertension and cancer. The leaves of the Madagascar periwinkle produce vinblastine and vincristine, used in cancer chemotherapy, ajmalacine, used as an antihypertensive, and serpentine, used as a sedative [1–3]. Antifeedant activity against herbivores and antimicrobial activity that inhibits fungal and bacterial growth have also been reported for *C. roseus* alkaloids such as vinblastine, catharanthine, ajmalacine and vindoline [4].

Periwinkle, as an indicator plant, is highly susceptible to phytoplasma and spiroplasma infection from different crops. It is commonly used as a source plant to maintain and study mollicutes because it is able to harbor many different known phytoplasmas and mollicutes can reach high titres in this species [5–11]. Periwinkle was the first non-rutaceous plant to have been found naturally infected by *Spiroplasma citri,* which is the type species of the genus *Spiroplasma* of the family Spiroplasmataceae, belonging to the class *Mollicutes* [12,13]. *Spiroplasma citri* can cause severe symptoms such as rapid decline in the number and size of the flowers, until flowering ceases, reduction in leaf size and chlorosis of leaf tips and margins, proliferation of auxillary buds, stunting and finally general yellowing that starts from the bottom and causes death to the infected periwinkle [14–17]. *Spiroplasma citri* is a motile, helically filamentous, mycoplasma-like organism that lacks a true cell wall [18] and infects the phloem sieve tubes of its hosts. It is in practice, an obligate parasite, surviving in citrus or in a variety of other host plants, with no saprophytic phase.

The mollicutes-plant interaction has been studied at the metabolite level, specifically sugar metabolism, revealing that they deplete sugar levels of the host plant [19–21]. Carbohydrates are the major sources of carbon and energy for spiroplasmas [22]. The phosphoenolpyruvate phosphotransferase system (PTS) is the major import system of carbohydrates in *S. citri*. Whilst *S. citri* is unable to use sucrose as anenergy source; fructose, glucose and trehalose are used, and fructose plays a key role in spiroplasmal pathogenicity [23,24].

The molecular mechanisms involved in the mollicute-plant interactions and the effects of phytoplasma and spiroplasma infections on gene expression of the host plants have been studied in the periwinkle, apricot (*Prunus armeniaca*), tomato (*Solanum lycopersicum*), poinsettia (*Euphorbia pulcherrima*) and grapevine (*Vitis vinifera*) [9,25–30]. Most of the gene expression studies have been carried out under controlled conditions, because combination of different biotic and abiotic environmental variables affect on field-grown plants which will result in a gene expression level inconsistency and might differ from those found under more controlled conditions. Therefore responses of plants at the transcriptional level to environmental change under natural field conditions are still poorly understood. In order to validate transcript analysis, experimentally and naturally *S. citri* infected, and healthy periwinkle plants, were compared at the gene expression level of extensin, metallothionein, heat shock protein 90, and strictosidine β-glucosidase genes.

Several defense-response proteins and pathogen-related proteins are induced by pathogen attack or by elicitor treatment. Hydroxyproline-rich glycoproteins (HRGPs), a class of defense proteins, are the major amino acid constituent in plant cell walls, and extensins are part of the large class of HRGPs. They are glycoproteins of the plant cell wall, characterized by their high hydroxyproline content and usually contain the repeating pentapeptide motif ser-Hyp4. They are thought to play essential developmental roles in primary wall biosynthesis, cell adhesion, the definition of cell morphology and the regulation of extension growth. The accumulation of HRGPs occurs in infected tissues, particularly in cell wall appositions, making the cell walls impermeable to pathogens, and is correlated with the expression of increased disease resistance [31]. They may act as structural barriers towards invading pathogens, provide a matrix for the deposition of lignin, and/or as specific agglutinins of microbial pathogens [31–33].

Metallothioneins (MT) are another class of defense proteins belonging to a superfamily of intracellular metal-binding proteins [34]. MTs are a family of low molecular weight, cysteine rich, metal binding proteins (MW 3500–14000 Da) [35,36]. Cysteine residues from MTs can capture harmful oxidant radicals like the superoxide and hydroxyl radicals. MTs reveal similar functional properties and are uniformly found in species ranging from microorganisms to mammals. MTs directly bind physiological metals such as zinc and copper, or indirectly, by binding xenobiotic heavy metals such as cadmium, mercury, and silver. The binding occurs via the thiol groups of the cysteine residues. MTs also have antioxidant activity. This property prevents metal ions from participating in the Fenton reaction, which is a primary source of highly reactive hydroxyl radicals in cells [35,37].

The term heat shock proteins (HSPs), also called stress proteins, refers to a set of proteins that are induced when a cell is exposed to elevated temperature or exposure to a variety of environmental stress conditions [2,38,39]. They also act as chaperones mediating the correct folding, assembly and transport of polypeptides or as proteases degrading the irreversibly unfolded proteins. The HSPs are present in all cells in all life forms, among prokaryotes and eukaryotes. They are located in the cytoplasm or in the endoplasmic reticulum, plastid and mitochondria. They have been classified into families based on their molecular weights, HSP100, HSP90, HSP70, HSP60, HSP40, HSP10 and alpha-HSPs or small heat shock proteins. HSP90 is one of the most important members in the chaperone family and is also widely distributed in plants [39–42].

β-glucosidases are hydrolase enzymes and related to sugar metabolism. Glycoside hydrolases have been classified into more than 100 families, based on amino acid sequence similarity. They have various functions in cell wall metabolism and lignification, signaling and hydrolysis of starch, activation of chemical defense compounds against plant pathogens and herbivores [43–47]. They are up-regulated in response to salt, cold and osmotic stress in Arabidopsis [48]. One of the major functions of β-glucosidases in higher plants is protection of plants against pathogens because of the pathogenesis-related proteins (PR proteins) and formation of bitter or toxic small molecules, such as cyanide, isothiocyanates, or DIMBOA (2,4-dihydroxy-7-methoxy-1,4-benzoxazin-3-one) from their corresponding glucosides [47,49–52]. β-glucosidases act as bio-activating components in activation of cyanogenic glucosidases, benzoxazinoid glucosidases, avenacosides and glucosinolates [41]. Strictosidine β-glucosidase (SGD) is an enzyme involved in terpenoid indole alkaloids (TIA) biosynthesis pathway of *C. roseus* which converts strictosidine to cathenamine and produces highly valuable pharmaceutical compounds [53]. Strictosidine β-*d*-glucosidase (SG), as the key enzyme, allows plants to synthesize the enormous variety of 2000 monoterpenoid indole alkaloids particularly in the three plant families Apocynaceae, Rubiaceae, and Loganiaceae [49,54,55]. Glucoside strictosidine and its deglucosylation product(s) formed by strictosidine β-*d*-glucosidase in *C. roseus* cells have a direct role in plant defense against several microorganisms through a plant protecting antimicrobial action [4]. In addition of this role, SGDs have a more direct role in plant defense related glucosidase as a damage-inducible biochemical defense system [49,56].

Only one study has been conducted at the gene expression level on *S. citri* infected periwinkle so far [9]. In this study, for the first time we describe triscript analysis of four selected genes involved in plant stress and defense responses by RT-qPCR following infection by *Spiroplasma citi.*,

## 2. Results and Discussion

**Figure 1:**
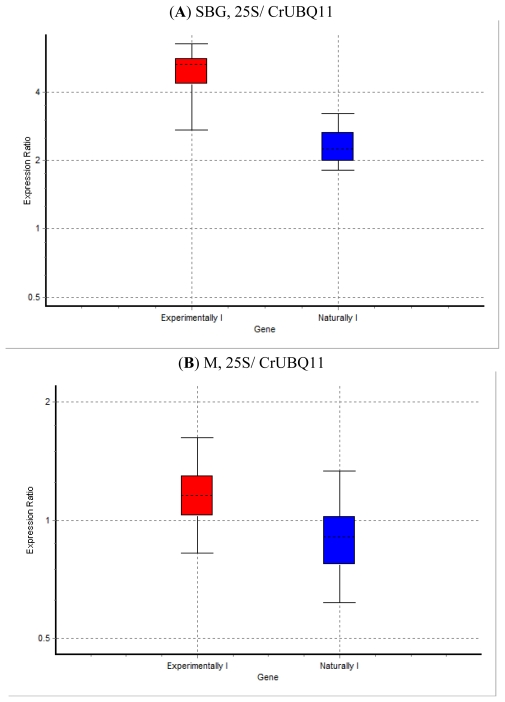

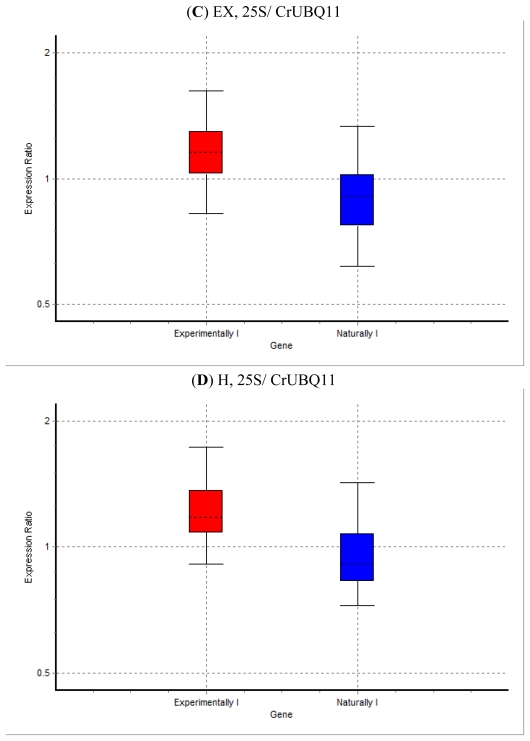
Relative expression levels of strictosidine β-glucosidase (SBG), metallothionein (M), extensin (EX) and heat shock protein (H) genes calibrated using 25S rRNA/CrUBQ11 reference genes in experimentally and naturally *S. citri* infected periwinkles by quantitative real-time PCR. (**A**) SBG, 25S/ CrUBQ11, *P*-value experimentally and naturally infected sample group: 0.002; (**B**) M, 25S/ CrUBQ11, *P*-value experimentally infected sample group: 0.076; *P*-value naturally infected sample group: 0.365; (**C**) EX, 25S/ CrUBQ11, *P*-value Experimentally infected sample group: 0.004; *P*-value Naturally Infected sample group: 0.248; (**D**) H, 25S/ CrUBQ11, *P*-value experimentally infected sample group: 0.091; *P*-value naturally infected sample group: 0.744.

RT-qPCR for the four defense response genes was performed to explore the variance of gene expression between healthy, and naturally and experimentally, *S. citri* infected periwinkle leaves. The expression profiles of metallothionein, heat shock protein 90, extensin and strictosidine β-glucosidase genes were compared using specific primers designed for periwinkle genes from sequences available in the GenBank, Database. The 25S rRNA and ubiquitin (UBQ11) reference genes of *Catharanthus roseus* were employed as expression control genes [57]. No significant differences were observed in metallothionein and HSP 90 between healthy and infected periwinkles. In the case of metallothionein and HSP 90, a similar level of expression was observed in healthy, and in naturally and experimentally spiroplasma-diseased periwinkles. A similar pattern of metallothionein, HSP 90 and strictosidine β-glucosidase expression was observed in experimentally and naturally infected periwinkle by quantitative real-time PCR. One difference from extensin expression was revealed by real-time PCR. The transcript level of extensin significantly increased in leaves of experimentally infected periwinkles compared to healthy ones. However, there were no significant differences in the relative expression of extensin in all naturally infected periwinkles compared with healthy ones by real-time PCR. Strictosidine β-glucosidase indicated significant upregulation in experimentally and naturally infected periwinkles. The relative expression of strictosidine β-glucosidase was higher in the experimentally infected, than in the naturally infected periwinkles as determined by RT-qPCR (Figure 1).

Metallothionein, heat shock protein, strictosidine β-glucosidase and extensin genes are related to plant defense and stress response. Changes in these genes expression to the spiroplasma infection were assessed using real-time PCR.

This result shows that the increase in cell wall hydroxyproline is a primary event in the host-pathogen interaction in spiroplasma infected periwinkle. In 2009, the repression of genes responsible for cell wall degradation and upregulation of genes involved in cell wall reinforcement have been found in Bois Noir phytoplasma infected grapevine [58]. They may act as structural barriers toward penetration and invading pathogens, provide a matrix for the deposition of lignin, and/or as specific agglutinins of microbial pathogens and could limit new infection and the spread of pathogen cells in the plant [31,32,58]. Extensin was up-regulated in experimentally spiroplasma-effected leaves while no significant difference in the relative expression was found in naturally infected leaves by real-time PCR. Thus, other environmental factors can effect extensin expression during infection and may suppress the expression of extensin.

In our study, up-regulation of strictosidine β-glucosidase in spiroplasma infected periwinkles support the result of other related research that indicated involvement of β-glucosidases and strictosidine β-glucosidase in plant defense and antimicrobial activity [4, 47, 49, 53, 56].

$$\text{strictosidine}+{\text{H}}_{2}\text{O}⇌D-\text{glucose}+\text{strictosidine aglycone }(\text{Wikipedia})$$

Strictosidine b-*d*-glucosidase hydrolyse strictosidine, which is the key intermediate enzyme in the biosynthesis of terpenoid indole alkaloids, to produce cathenamine and subsequently important pharmaceutically alkaloids which are also toxic for pathogens [54,55]. Therefore they play a protective role against pathogen attack.

Although there was a difference in the expression level between experimentally and naturally infected periwinkles, the relative expression of Strictosidine β-glucosidase showed slightly higher expression in the experimentally spiroplasma-inffected leaves than in the naturally infected leaves. It reveals other environmental factors can affect on Strictosidine β-glucosidase expression. Hence, a gene expression study should be done under controlled conditions. Otherwise, expression of certain genes would be affected by biotic and abiotic stresses, and other environmental factors. Lower expression in naturally infected, compared to experimentally infected, plants support the result of other related research that showed the same genes in herbicide treated wheat, as under controlled conditions, were up-regulated in the field at a weaker level [59].

No significant changes in expression were observed in metallothionein and HSP90 expression. HSP90 was equally expressed both in healthy and infected periwinkles. It is possibly not involved in the plant response to spiroplasma infection. Although, metallothionein expression level was elevated in infected leaves, it was not significantly different from healthy leaves. Significant changes were also not observed for HSP70 expression in phytoplasma infected grapevine [28], while over-expressed of HSP70 and metallothionein were reported in phytoplasma infected leaves of apricots by differential display technique [26].

## 3. Experimental Section

### 3.1. Plant Material

Naturally *Spiroplasma citri* infected Madagascar periwinkles *(Catharanthus roseus* Don. L. G.) were collected in Serdang, Malaysia. Periwinkles were grown from seed in jiffy peat pellets. One month old seedlings were then transplanted to pots containing sterilized soil. For gene expression studies, periwinkles were inoculated with *S. citri* by side grafting of naturally infected periwinkle. *Spiroplasma citri* grafted and healthy periwinkles were maintained in the greenhouse. At 6 weeks after inoculation, symptomatic leaves were harvested from artificially infected individuals for the post-symptomatic gene expression at the transcriptional level, albeit for the naturally infected plants it is impossible to know when they actually became infected.

### 3.2. *Spiroplasma citri* Detection

The infection of naturally and experimentally (artificially) infected periwinkles by *S. citri* were confirmed by spiroplasma symptoms and PCR using the spiroplasma specific primer set ScR16F1/ScR16R1 [60].

### 3.3. RNA Isolation

Leaves of naturally and experimentally infected and healthy periwinkle (150 mg) were frozen in liquid nitrogen and homogenized to a fine powder using a cold mortar and pestle. Total RNA was extracted from leaves using the CTAB method [61] and treated with DNaseI to remove any traces of DNA, following the manufacturer’s protocol (QIAGEN, Hilden, Germany). Finally RNA was eluted with 50 μL of RNase-free water and purified by NucleoSpin Clean-up (Macherey-Nagel). The RNA concentrations were accurately determined by a Nanodrop ND-1000 spectrophotometer (NanoDrop Technologies) with absorbance at 260 nm and agarose gel electrophoresis stained with ethidium bromide to verify the quality.

### 3.4. Primer Designing

Sequences of periwinkle defense response genes include metallothionein, heat shock protein 90, extensin and strictosidine β-glucosidase were obtained from NCBI and specific primer for each enzyme was designed using Primer3 software under the default parameters (http://primer3.sourceforge.net/) [62] (Table 1).

### 3.5. Real-Time PCR Analysis

The SYBR green I one-step real-time RT-PCR assay was carried out in a 96-well plate in a final volume of 25 μL to evaluate the expression pattern of four defense response genes in spiroplasma infected periwinkles. The real-time PCR mixture contained 1 μL of RNA, 12.5 μL of 2× Quantifast SYBR Green RT-PCR Master Mix (Qiagen, Hilden, Germany) which includes ROX as a passive dye and 1 μM of each gene-specific primer. The real-time PCR reactions were performed using iCycleriQ4 (Bio-Rad, USA) under the following conditions: 10 min at 50 °C, 5 min at 95 °C, followed by 40 cycles of 10 s at 95 °C and 30 s at 60 °C. Melting curve (disassociation) analysis (60–95 °C) was done to verify amplicon specificity after 40 cycles. Normalization was performed employing 25S rRNA (25S rRNAF: 5′-CCAGGCCCCGATGAGTAGGA-3′ and 25S rRNAR: 5′-TTTCCCCTCTTCGGCCTTC-3′) and CrUBQ11 (forward primer: 5′-GGAAGGCATTCCACCAGACCA-3′ and reverse primer: 5′-TACCTC CCCGGAGACGAAGC-3′) periwinkle reference genes [57]. In a 96-well plate, each sample was analyzed in duplicate. All data in this study were obtained from four independent biological replicates. PCR efficiency of all reactions was between 89 and 98%.

Data analysis of qRT-PCR results was performed using REST software (Qiagen, Hilden, Germany). The relative gene expression was quantified according to the manufacturer’s instructions. Difference between treatments was considered as statistically significance when *P* < 0.05.

## 4. Conclusions

These results provide evidence that strictosidine b-*d*-glucosidase and extensin are involved in the defense response in periwinkle against *S. citri* infection. A gene expression study should be performed under both controlled and natural field conditions to validate gene expression results, because expression of certain genes would be affected by other biotic and abiotic environmental factors. Expression of strictosidine β-glucosidase in response to *S. citri* infection was fully consistent in the infected plants, regardless of natural or artificial infection; hence it is proven to be feasible as a host biomarker. Therefore strictosidine β-glucosidase is a potential biomarker that can be further tested for early expression. It is also a useful gene in the germplasm for improvement of the cultivars in breeding programs. This result could provide new insights into further understanding of the disease-tolerance mechanism in periwinkle. It may play significant roles in enhancing disease tolerance through increase of the TIAs level in *C. roeseus* which would also be an issue for further investigation.

## Figures and Tables

**Table 1:** Specific primers employed in this study.

primer	Nucleotide sequence (5′-3′)	Size of PCR products (bp)	Accession number	Target gene	Reference
MF	CATGTCTTGCTCCTGTGGTG	175	DQ016341	metallothionein	in this study
MR	ATGTCCTCCTTCTGCTCCAA				
HSPF	CGGCTCATGTACCAGACCGCA	168	L14594	heat shock	in this study
HSPR	TGTGCCGGATTCAGCCTCAGC			protein 90	
EXF	CTCCACCATCAGTCCACAAA	181	D86853	extensin	in this study
EXR	GGAGTGGGTGGGGGATATT				
BGF	TCACAAAGCTGCTGTGGAAG	182	AF112888	β-glucosidase	in this study
BGR	CACCCGTTGTTAATGGCTCT				
